# Regular Exercise Influences Sleep Patterns in Depressed Individuals

**DOI:** 10.1192/j.eurpsy.2025.2332

**Published:** 2025-08-26

**Authors:** J. Kim, S. Jaka, T. Shah, S. Gunturu, O. El Shahawy

**Affiliations:** 1 BronxCare Health System, Bronx; 2 New York University Grossman School of Medicine, New York, United States

## Abstract

**Introduction:**

Physical activity has been associated with improved sleep health, yet the specific impact on individuals with depressive disorders remains underexplored. This study aims to investigate the relationship between regular physical exercise and sleep duration in individuals diagnosed with a depressive disorder, utilizing data from the Behavioral Risk Factor Surveillance System (BRFSS). Understanding this relationship could inform integrative approaches to managing depressive symptoms and improving overall sleep health in this population.

**Objectives:**

- To analyze the impact of regular physical exercise on sleep duration among individuals with a depressive disorder.
- To assess the potential role of physical exercise in mitigating inadequate and prolonged sleep patterns within this population.

**Methods:**

Data from the BRFSS for the years 2013, 2014, 2016, 2018, 2020, and 2022 were analyzed to explore the relationship between physical exercise and sleep duration in individuals with depressive disorders. The study included 518,214 participants who reported having a depressive disorder. Of these, 342,276 (weighted 68.5%) reported engaging in physical exercise within the past 30 days. Sleep duration was categorized, and regression analysis was used to assess the association between recent physical exercise and sleep duration.

**Results:**

The analysis indicated that individuals with a depressive disorder who engaged in physical exercise in the past 30 days were less likely to experience inadequate sleep (6-7 hours, Odds Ratio [OR] = 0.83, p < 0.05) and more likely to achieve adequate sleep (7-9 hours, OR = 1.18, p < 0.001) compared to those who did not exercise. They were slightly less likely to have prolonged sleep (9-12 hours, OR = 0.9, p < 0.05). No significant associations were found for very inadequate sleep (<6 hours, OR = 1.34) and very prolonged sleep (>12 hours, OR = 0.6) with physical exercise (p > 0.05).

**Image 1:**

**Figure:**
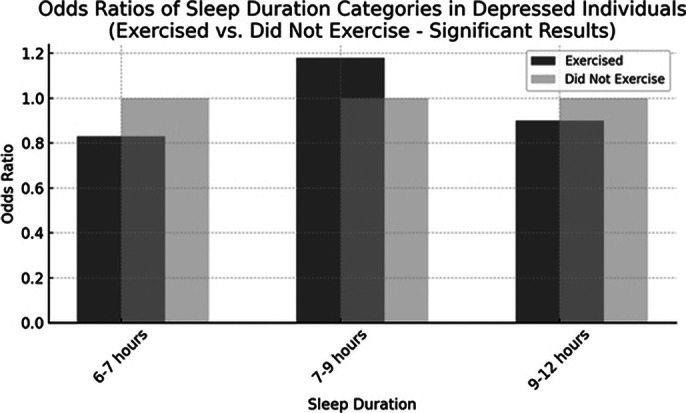

**Conclusions:**

Regular physical exercise appears to be associated with better sleep outcomes in individuals with depressive disorders, particularly in promoting adequate sleep duration (7-9 hours). Exercise was linked to a reduced likelihood of inadequate sleep (6-7 hours) and a slight decrease in prolonged sleep (9-12 hours). However, no significant associations were found for very short (<6 hours) or very prolonged (>12 hours) sleep durations. These findings highlight the potential role of physical exercise in managing sleep health within this population. Incorporating regular physical activity into treatment plans for depression may improve sleep quality and contribute to better overall health outcomes. Further research is needed to understand the mechanisms and long-term effects of exercise on sleep patterns in those with depressive disorders.

**Disclosure of Interest:**

None Declared

